# Chimney Stove Intervention to Reduce Long-term Wood Smoke Exposure Lowers Blood Pressure among Guatemalan Women

**DOI:** 10.1289/ehp.9888

**Published:** 2007-02-14

**Authors:** John P. McCracken, Kirk R. Smith, Anaité Díaz, Murray A. Mittleman, Joel Schwartz

**Affiliations:** 1 Department of Epidemiology and; 2 Department of Environmental Health, Harvard School of Public Health, Boston, Massachusetts, USA; 3 Environmental Health Sciences Division, School of Public Health, University of California, Berkeley, California, USA; 4 Center for Health Studies, Universidad del Valle, Guatemala City, Guatemala; 5 Cardiovascular Epidemiology Research Unit, Beth Israel Deaconess Medical Center, Boston, Massachusetts, USA

**Keywords:** biomass fuel, cardiovascular disease, echo-intervention, indoor air pollution, RESPIRE project

## Abstract

**Background and Objective:**

RESPIRE, a randomized trial of an improved cookstove, was conducted in Guatemala to assess health effects of long-term reductions in wood smoke exposure. Given the evidence that ambient particles increase blood pressure, we hypothesized that the intervention would lower blood pressure.

**Methods:**

Two study designs were used: *a*) between-group comparisons based on randomized stove assignment, and *b*) before-and-after comparisons within subjects before and after they received improved stoves. From 2003 to 2005, we measured personal fine particle (particulate matter with aerodynamic diameter < 2.5 μm; PM_2.5_) exposures and systolic (SBP) and diastolic blood pressure (DBP) among women > 38 years of age from the chimney woodstove intervention group (49 subjects) and traditional open wood fire control group (71 subjects). Measures were repeated up to three occasions.

**Results:**

Daily average PM_2.5_ exposures were 264 and 102 μg/m^3^ in the control and intervention groups, respectively. After adjusting for age, body mass index, an asset index, smoking, secondhand tobacco smoke, apparent temperature, season, day of week, time of day, and a random subject intercept, the improved stove intervention was associated with 3.7 mm Hg lower SBP [95% confidence interval (CI), −8.1 to 0.6] and 3.0 mm Hg lower DBP (95% CI, −5.7 to −0.4) compared with controls. In the second study design, among 55 control subjects measured both before and after receiving chimney stoves, similar associations were observed.

**Conclusion:**

The between-group comparisons provide evidence, particularly for DBP, that the chimney stove reduces blood pressure, and the before-and-after comparisons are consistent with this evidence.

Approximately half of the world’s households depend on biomass (e.g., wood, crop residues, and animal dung) and coal for cooking and heating (Smith et al. 2004). Most of this solid fuel use occurs in developing countries, where poor households generally use open fires or inadequately vented stoves. These fuel-stove combinations result in high indoor levels of fine combustion-generated particles and other pollutants. As part of the World Health Organization’s (WHO) Comparative Quantification of Health Risks study, Smith and colleagues (2004) estimated that 1.6 million premature deaths and 3.6% of the global burden of disease were attributed to indoor air pollution from the use of solid fuels.

Although numerous epidemiologic investigations have concluded that fine ambient particles and secondhand tobacco smoke (SHS) are associated with cardiovascular morbidity and mortality (Brook et al. 2003, 2004; Dockery et al. 1993; He et al. 1999; Pope et al. 1995; Schwartz 1993, 1994; Thun et al. 1999), the WHO risk assessment attributes only respiratory diseases to household solid fuel use (Smith et al. 2004). The discrepancy derives from the lack of epidemiologic studies of the cardiovascular impacts of indoor wood smoke. Although there may be important toxicologic differences among ambient air pollution, SHS, and indoor air pollution from biomass combustion, the body of evidence on the first two, combined with the toxicologic properties of wood smoke (Naeher et al. 2007), raises concern about the potential cardiovascular effects of indoor wood smoke.

In the Western Highland region of Guatemala, 24-hr average concentrations of respirable particles [particulate matter (PM) < 3.5 μm in aerodynamic diameter] were 1,930 μg/m^3^ in homes where open fires were used for cooking (Albalak et al. 2001). However, studies have demonstrated that the *plancha*, an improved woodstove with a chimney, reduces kitchen air pollutant levels and personal exposures (Albalak et al. 2001; Bruce et al. 2004; McCracken and Smith 1998; Naeher et al. 2000; Smith et al. 1993).

We participated in RESPIRE (Randomized Exposure Study of Pollution Indoors and Respiratory Effects), which used the *plancha* to conduct the first randomized intervention to reduce long-term air pollution exposures (Smith et al. 2006). This trial focused on respiratory outcomes, but created an opportunity to study the potential cardiovascular health benefits of reduced air pollution exposures.

Elevated blood pressure (BP) predicts cardiovascular morbidity and mortality, and reductions in BP have been shown to reduce risk (Glynn et al. 2002; Sesso et al. 2000, 2003). Several studies have observed positive associations between ambient air pollutants and BP (de Paula Santos et al. 2005; Ibald-Mulli et al. 2001; Linn et al. 1999; Zanobetti et al. 2004), although other studies have failed to find associations or have even detected inverse associations between ambient air pollutants and BP (Brauer et al. 2001; Ibald-Mulli et al. 2004). Moreover, ambient air pollution studies examined short-term changes in BP, alhough it is only long-term differences in BP that have clear health consequences.

We hypothesized that the *plancha* improved woodstove would be associated with long-term reductions in systolic blood pressure (SBP) and diastolic blood pressure (DBP) among healthy adults. As supporting evidence, we also aimed to determine whether the stove intervention was associated with reduced personal exposures to fine particulate air pollution.

## Materials and Methods

The protocols for the stove intervention trial were approved by the human subjects committees at the University of California-Berkeley, the U.S. Centers for Disease Control and Prevention, Liverpool University (UK), and the Universidad del Valle in Guatemala. The cardiovascular substudy was approved by the human subjects committees of the Harvard School of Public Health and Universidad del Valle, and all participants gave informed consent before data collection.

### Study site

San Marcos, Guatemala, borders the Pacific Ocean to the west and Chiapas, Mexico, to the north. From the rural area within this district, near the towns of San Lorenzo and Comitancillo, 23 villages were chosen based on a rapid assessment showing high prevalence (> 90%) of cooking with open wood fires. The study villages lie at 2,200–3,000 m elevation, creating a cool climate that necessitates enclosed homes, leading to high indoor air pollution concentrations.

### Study population in randomized trial

Household eligibility criteria for the randomized trial, which focused on acute lower respiratory infections in infants, included exclusive use of an open biomass fire for cooking and having a pregnant woman or infant < 4 months of age. Households (*n* = 537) were recruited and randomized from October 2002 through May 2003.

### Stove intervention

Before intervention, all recruited households used open fires at or near kitchen-floor level without a chimney. The intervention—an improved woodstove called the *plancha*—has been widely disseminated in Guatemala. Key features of the stove are a cooking surface at waist height, an enclosed combustion chamber, and a chimney to vent emissions from the kitchen. An outer wall of concrete blocks and bricks is filled partly with earth, on top of which the firebrick combustion chamber is formed. The cooking surface is a metal plate used for making tortillas and has three potholes with concentric rings and a center disk that can be removed to place a pot directly over the fire. The front of the stove has a door for fuel feeding, and the chimney is attached to the back of the combustion chamber. The stoves were constructed by Tasprovi, a masonry contractor from Quetzaltenango, Guatemala, which also provided stove repair during the trial period.

After administration of a baseline questionnaire, randomization was carried out within blocks of 10 neighboring households. The intervention group received improved *plancha* stoves, and the control group continued using the traditional open fires for cooking. The control households received the improved stove at the end of the trial period, which we refer to as the echo-intervention.

### Cardiovascular substudy population

After randomization and stove installation, we recruited women > 38 years of age living in households of the main study in 18 villages closest to the study headquarters. The baseline questionnaire provided a list of 238 women (115 control, 123 intervention), among whom 208 (104 control, 104 intervention) were living and found at home during the substudy recruitment visit. The additional eligibility criteria, which were met by 185 (95 control, 90 intervention) of these women, were that they cooked daily and resided in a study house at the time of recruitment. Among the 185 eligible women invited to participate, the response rates were 75% among the control households and 54% among the intervention households, resulting in 120 (71 control, 49 intervention) participants overall. None of these women were pregnant, had given birth during the previous three months, nor were breast-feeding at the time of the study. Most were grandmothers of a child in the main study.

### Personal PM_2.5_ measurements

We measured average personal PM_2.5_ (suspended PM < 2.5 μm in diameter) with gravimetric samplers during the 24 hr before blood pressure measurements. Each air sampler setup included an Apex pump (Casella Inc., Bedford, UK), a Triplex Sharp-Cut Cyclone (BGI Inc., Waltham, MA, USA), and a 37-mm Teflon filter placed on top of a drain disc and inside a metal filter holder. The flow rate was set to 1.5 L/min and measured at the start and end of sampling with a soap bubble flow meter (Gilian Inc., West Cladwell, NJ, USA). The air sampler, weighing about 0.5 kg, was carried in a shoulder bag with the inlet clipped to the strap above waist height. The participants were instructed to carry the bags everywhere they went and place them near their bed when sleeping. The filters were weighed with a micro-balance (Mettler-Toledo Inc., Columbus, OH, USA) under atmosphere-controlled conditions before and after sampling.

### Blood pressure measurements

Participants were transported in project vehicles from their homes to the San Lorenzo Health Center, where all BP measures were taken between 1400 and 1800 hr. An automatic blood pressure monitor (52000 series; Welch Allyn, Skaneateles Falls, NY, USA) was used to measure SBP and DBP in the supported right arm of the seated subject after 10-min rest. Three repeat measures were taken within a 10-min period of continued rest.

Although 10 control subjects and 7 intervention subjects dropped out of the study or moved away from the study site after one measurement round, the protocol was repeated on two or three occasions for most participants. The trial period, from July 2003 through December 2005, included 111 measures among 71 women in the control group cooking over open fires and 115 measures among 49 women in the intervention group using the improved stoves. On average, the intervention participants had been cooking with the *plancha* for 293 days (range, 2–700 days). After control households received the echo-intervention, an additional 65 measures were taken among 55 control subjects from March 2004 through March 2005. On average, the post–echo-intervention measures were taken 63 days (range, 0–342 days) after control households started using the improved stoves.

### Statistical analysis

We tested the baseline characteristics of the BP study intervention and control groups for comparability using *t*-tests for continuous variables and chi-square tests for binary variables in SAS version 9.1 (SAS Institute Inc., Cary, NC, USA).

We used the average of the second and third of three consecutive BP measures taken during each visit as the estimate for that day. SBP and DBP were the dependent variables in separate regression analyses. We estimated the intervention effects using two different study designs: between-group comparisons based on randomized stove assignment, and before-and-after echo-intervention comparisons among the control group.

#### Between-group comparisons

The between-group study design compares BP between control and intervention groups during the trial period, when control homes had open fires for cooking and intervention homes had improved stoves. To illustrate the distributions of personal PM_2.5_ exposure and BP by study group, we plotted smoothed probability densities with spans determined by the default rule of thumb (nrd0) using R 2.4.0 software (The R Foundation of Statistical Computing; www.r-project.org).

Mixed models were run using the linear mixed effects (lme) function in R software. The model for the between-group estimates of the effect of improved stoves on BP is


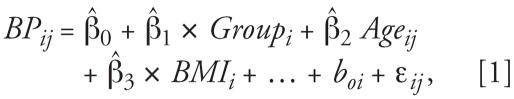


where *i* denotes subject and *j* denotes the repeated measures within subjects. *BMI* is body mass index. β̂_1_ is the effect estimate for the randomly assigned improved stove (*Group*), which has only the subscript *i* because it varies between people but not temporally. β̂_2_, β̂_3_, and so on represent the effects of covariates, the *b_oi_* term is a random intercept for the *i*^th^ subject, and ɛ*_ij_* are the residuals. The random intercept accounts for correlation among repeated measures and therefore provides appropriate weighting of information from each subject, even though the number of measures per subject may vary.

Although randomization made the two groups similar according to all baseline covariates measured, because of the small sample size and potential for differential selection by intervention status and predictors of BP, we adjusted for covariates that may predict BP between subjects, such as age, BMI, ever smoking, SHS exposure, use of a *temascal* (wood-heated sauna), having household electricity, and an asset index as a measure of socioeconomic status. Age and BMI were fit as linear terms. We used binary indicator variables for smoking, SHS exposure, *temascal* use, and household electricity. The asset index is the sum of binary indicators for having a bicycle, a radio, and a television, and was entered as categorical variable. To increase precision, we also considered time-varying covariates, such as apparent temperature, season, day of the week, and time of day. We used linear terms to control for daily average apparent temperature and time of day and dummy variables for each day of the week and for rainy (1 May–31 October) versus dry season (1 November –30 April).

#### Before-and-after comparisons

The before-and-after study design estimates the within-subject effect of the stove intervention on BP by comparing the same people before and after adoption of the chimney stove. Distributions of personal PM_2.5_ and BP among the control group during the trial and echo-intervention periods were illustrated using smoothed probability densities as described above. Only control subjects who had echo-intervention measures are included in these analyses, which were based on the following mixed model:


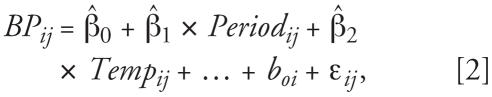


where the stove variable (*Period*), an indicator for the echo-intervention period, now also has a subscript *j* to denote that it varies across measurements within subjects. *Temp* refers to daily average apparent temperature. Again, a random intercept for subject is included.

In the before-and-after models, people serve as their own controls, so the estimates for the effect of the echo-intervention cannot be biased by any factors that do not vary temporally. However, within-subject analyses do compare BP measures at different times and are therefore susceptible to confounding by time-varying covariates, which is why we adjusted for age, season, day of the week, time of day, and daily average apparent temperature. To be consistent with the between-group model and possibly further improve efficiency, we adjusted for BMI, an asset index, *temascal* use, household electricity, ever smoking, and SHS exposure. All covariates were entered in the model in the same form as in the between-groups analyses.

#### Sensitivity analyses

SBP normally rises with age and often levels off around 70 years of age, though the point at which the association flattens appears to differ by region (Lawes et al. 2004). DBP has been found to increase until about 60 years of age, after which it declines with increasing age (Franklin et al. 1997). As sensitivity analyses, we used penalized regression splines in generalized additive mixed models to adjust for the potential nonlinear relationships between BP and age. This was performed for both the between-group and before-and-after analyses using the generalized additive mixed-model (gamm) function in R software (Lin and Zhang 1999).

Effect modification by smoking status and SHS exposure was tested by creating interaction terms between these variables and the stove type indicators in the between-group and before-and-after analyses.

## Results

### Between-group comparisons

Table 1 compares the baseline characteristics of participants and nonparticipants among all women classified as eligible based on the RESPIRE baseline questionnaire. The average age of the women, household elevation, and the proportions having various assets and exposures were remarkably similar among participants and nonparticipants. There was no important or statistically significant difference for any measured baseline covariate, providing evidence that selection bias was unlikely.

Table 2 shows the baseline characteristics among participants randomized to the control and intervention groups. Age and BMI, two important predictors of BP, were very close on average. Several indicators of socioeconomic status were also compared without finding any large differences. Similarly, the percentages of women who reported ever smoking or other sources of household air pollution, such as wood-fired saunas, kerosene lamps, and SHS exposure, were also very similar.

**Figure 1:**
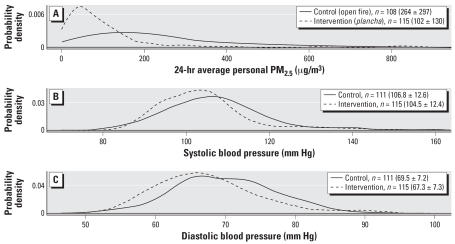
Between-group comparisons. Smoothed probability densities by randomized stove group (control, *n* = 71; intervention, *n* = 49) during the trial period (June 2003 through December 2004) of (*A*) 24-hr average PM_2.5_; (*B*) SBP; and (*C*) DBP. Mean ± SD for each distribution shown in parentheses next to number of repeated measures (*n*).

Figure 1A–C shows the smoothed probability densities of PM_2.5_, SBP, and DBP, respectively, by study group during the trial period. Personal PM_2.5_ was about 61% lower on average and had a narrower distribution among the *plancha* improved stove group compared with the open fire group. The lower particle exposures were accompanied by noticeable downward shifts in the SBP and DBP distributions in the intervention group using the improved *plancha* stove.

The crude and adjusted mixed-model estimates of between-group differences in mean SBP and DBP are shown in Table 3. After adjustment, the *plancha* improved stove was associated with 3.7 mm Hg lower SBP [95% confidence interval (CI), −8.1 to 0.6] and 3.0 mm Hg lower DBP (95% CI, −5.7 to −0.4), both similar to the unadjusted associations. Excluding smokers did not alter the results, and there was no significant interaction between intervention and ever smoking nor intervention and SHS exposure for either BP measure (all *p* > 0.25).

Using penalized splines to adjust for age, we found a significant nonlinear relationship between SBP and age (*p* < 0.001), and the association of SBP with the chimney stove in the between-group model increased in magnitude and precision to a reduction of 4.0 mm Hg (95% CI, −8.1 to 0.2; *p* = 0.06). DBP did not have a significant nonlinear relationship with age (*p* = 0.62), and the estimate of the stove effect on DBP was unchanged by adjustment for age using the penalized spline model.

### Before-and-after comparisons

**Figure 2:**
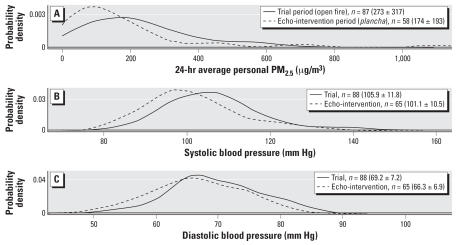
Before-and-after comparisons. Smoothed probability densities for control group by study period (trial period June 2003 through December 2004, echo-intervention period March 2004 through March 2005) of (*A*) 24-hr average PM_2.5_; (*B*) SBP; and (*C*) DBP. Mean ± SD for each distribution shown in parentheses next to number of repeated measures (*n*).

Figure 2A–C shows the smoothed probability densities of PM_2.5_, SBP, and DBP, respectively, among the control group during the trial period and after the echo-intervention. Compared with use of open fires for cooking, personal PM_2.5_ was reduced by about 38% on average and had a narrower distribution when the same subjects used the *plancha* improved stove. The reduced particle exposures were accompanied by noticeable downward shifts in the SBP and DBP distributions the improved *plancha* stove was used.

The crude and adjusted mixed-model estimates of before-and-after differences in mean SBP and DBP are shown in Table 4. In the adjusted model, the echo-intervention was associated with a 3.1 mm Hg decline in SBP (95% CI, −5.3 to −0.8) and a 1.9 mm Hg decline in DBP (95% CI, −3.5 to −0.4). Excluding smokers did not alter the results, and there was no significant interaction between stove and ever smoking nor stove and SHS exposure for either BP measure (*p* > 0.25).

In the before-and-after study design, adjustment for nonlinear associations between BP and age using penalized splines did not alter the associations of stove with either BP measure.

## Discussion

SBP and DBP were lower among women randomly assigned to cook with the *plancha* improved stove instead of the traditional open fire. After adjustment, the 95% CI for the mean difference in DBP did not overlap zero, and the 95% CI for the mean difference in SBP only barely overlapped zero. A few subjects from both groups were measured each week during the trial period, making any unmeasured time-varying covariates unlikely to be strong confounders. Adjustment for predictors of BP in the between-group model moved the effect estimates further away from the null. Similar estimates from the between-group and before-and-after analyses greatly strengthen the evidence because these are not susceptible to the same sources of bias. That participants and research assistants were not blinded to exposure status is unlikely to induce bias, because automatic blood pressure monitors were used. BP differences associated with stove type were estimated with greater precision in the before-and-after than in the between-group analyses, despite the larger sample size in the latter. This is attributed to lower random variability in BP within-subjects than in between-subjects.

Although past indoor air pollution studies in rural Guatemala have measured kitchen levels of particles and personal levels of gases (Albalak et al. 2001; Bruce et al. 2004; McCracken and Smith 1998; Naeher et al. 2000; Smith et al. 1993), this is the first such study to measure personal particle exposures. The substantial reduction in personal PM_2.5_ exposure associated with *plancha* compared with open fire use strengthens the evidence that the observed BP differences may have been caused by reduced wood smoke exposure. These personal exposures among both open fire and *plancha* users were much lower than average kitchen levels found in similar populations with the same stove types (Albalak et al. 2001). In addition, the proportional personal exposure reduction associated with the *plancha* was less than that reported for kitchen levels. These differences are consistent with typical time–activity patterns, because women do not spend all day in the kitchen. Despite the intervention, exposures remained well above the WHO Air Quality Guidelines for indoor and outdoor particle exposures (WHO 2005).

At much lower concentrations than the wood smoke exposures we observed, ambient PM has been found to be associated with BP among the general population, people with severe chronic obstructive pulmonary disease, and cardiac rehabilitation patients (Ibald-Mulli et al. 2001; Linn et al. 1999; Zanobetti et al. 2004). There is evidence of both immediate effects (Urch et al. 2005) and delayed effects over several days (Zanobetti et al. 2004). Oxidative stress and systemic inflammation are central to leading mechanistic hypotheses for effects of fine particles on cardiovascular health (Brook et al. 2004). Evidence of increased reactive oxygen species concentrations in the heart and lungs of rats exposed to concentrated ambient particles supports this hypothesis (Gurgueira et al. 2002). Providing a mechanistic link to blood pressure, endothelin-1, which modulates systemic vascular tone, is known to increase in response to reactive oxygen species (Haynes and Webb 1998) and urban PM (Bouthillier et al. 1998). Li and colleagues (2005) found that losartan, an antagonist of angiotensin II type 1 receptors, inhibits the vasoconstriction effect of urban particles on human pulmonary artery endothelial cells. In addition to its effects on blood pressure, angiotensin II is also a proinflammatory mediator (Ridker et al. 2006), raising the possibility that the local renin–angiotensin system and systemic inflammation may be interrelated components of the cardiovascular system’s response to ambient particles and the oxidative stress they induce.

Supporting the biologic plausibility of the effect of wood smoke exposure itself on blood pressure, a controlled experiment found increased systemic inflammation markers, blood coagulation factors, and lipid peroxidation after wood smoke exposure among healthy adults (Barregard et al. 2006). In particular, increased serum amyloid A and urinary excretion of F_2_-isoprostane the day after 4-hr wood smoke–PM_2.5_ exposures in the range 240–280 μg/m^3^ shows that measurable oxidative stress and systemic inflammation result from short-term exposure at levels similar to the daily averages we observed among women cooking over open fires.

### Limitations

Although the women were recruited from randomized groups, the proportion of eligible women participating was differential by study group (75% for control group and 54% for intervention group). If participation was also associated with BP, selection bias may have produced study groups that were not comparable. Although the original study design included baseline BP measures, ethical approval was given after the intervention group received improved stoves. Adjustment for age, socioeconomic indicators, and BMI is likely to have at least partially attenuated the potential selection bias. Furthermore, we did not find any meaningful difference between the groups out of all available baseline covariates. 

The before-and-after comparisons rely on the assumption that BP would not have changed in the control group in the absence of the echo-intervention. There may be unmeasured time-varying covariates that influence BP and were associated with the echo-intervention. In addition, because not all control subjects measured during the trial period were also measured during the echo-intervention period, it is possible that we unintentionally selected people for the before-and-after comparisons who happened to have higher than usual BP before the echo-intervention. The trial period BP among the 16 control subjects excluded from the before-and-after comparisons due to missing post-echo-intervention measures (SBP = 110.2 and DBP = 71.0 mm Hg; *n* = 23) were higher than the trial period BP among the 55 subjects measured both before and after (SBP = 105.9 and DBP = 69.2 mm Hg; *n* = 88). Thus, there is no evidence of spurious findings due to selection of subjects for the echo-intervention follow-up who happened to have higher blood pressure when measured during the trial period.

Although this work provides evidence that cardiovascular effects associated with introduction of the improved stove are likely caused by reduced air pollution exposures, the intervention may alter other factors that influence BP, such as diet and activity patterns. The analysis does not address what component of the combustion products reduced by the improved stove is most likely to be associated with these reductions; the presentation of PM_2.5_ mass reductions is just meant to illustrate that the stove intervention had an effect, and does not imply that mass is more important than particle number, area, or composition. The main focus here is on estimating the effect of a randomly assigned intervention, and exposure–response analyses using personal fine particles and carbon monoxide as the exposures will be presented separately.

### Summary and implications

To our knowledge, this is the first study to examine cardiovascular health effects of wood smoke from household stoves, and the first use of a randomized intervention to assess cardiovascular effects of particles. A decrease in BP associated with use of chimney stoves instead of open fires contributes to the understanding of cardiovascular effects of long-term wood smoke exposure. This study suggests that, in addition to respiratory disease, cardiovascular disease may be a component of the public health burden of indoor air pollution in developing countries. More than 2 billion people rely on solid biomass fuels (e.g., wood, animal dung, and crop residues) for cooking and heating (Smith et al. 2004), but wood smoke exposure is not limited to rural areas of developing countries. Household use of wood fuel contributes significantly to ambient particle levels in many areas of the developed world and has been estimated to be the largest source in some areas (Naeher et al. 2007). Further research should aim to determine whether wood smoke is a cause of clinical cardiovascular disease.

## Figures and Tables

**Table 1:** Baseline data comparing women > 38 years of age from the RESPIRE-Guatemala trial by whether they participated in the blood pressure study.

Characteristic	Participants (*n* = 120)	Nonparticipants (*n* = 118)	*p*-Value^a^
Age (years)^b^	53.3 ± 12.0	51.2 ± 11.4	0.16
Household characteristics			
Elevation (m)	2,630 ± 187	2,609 ± 184	0.39
Kitchen volume (m^3^)	45.6 ± 22.8	49.0 ± 20.9	0.24
Electricity in house (%)	72	78	0.26
Radio (%)	93	90	0.37
Television (%)	25	25	0.94
Bicycle (%)	28	23	0.34
Cows (%)	53	49	0.55
Horses (%)	40	39	0.89
Sheep (%)	54	47	0.25
Other air pollution sources (%)			
*Temascal* wood-fired sauna	93	91	0.45
Secondhand tobacco smoke	28	25	0.61

a Two-sided *p*-values from *t*-tests for continuous variables and chi-square tests for categorical variables.

b Values are mean ± SD for continuous variables.

**Table 2:** Baseline data comparing women in the RESPIRE-Guatemala blood pressure study by randomized stove group.

Characteristic	Control (*n* = 71)	Intervention (*n* = 49)	*p*-Value^a^
Personal			
Age (years)^b^	52.6 ± 11.1	54.1 ± 11.1	0.48
Body mass index (kg/m^2^)	24.3 ± 3.0	24.8 ± 3.2	0.39
Ever smoked (%)	11	10	0.85
Household			
Elevation (m)	2,617 ± 189	2,649 ± 183	0.35
Kitchen volume (m^3^)	47.2 ± 22.9	43.6 ± 22.8	0.42
Electricity in house (%)	73	69	0.65
Radio (%)	93	92	0.82
Television (%)	21	31	0.24
Bicycle (%)	27	31	0.65
Asset index^c^ (%)			
0	6	8	0.50
1	55	45	
2	32	33	
3	7	14	
Cows (%)	54	51	0.80
Horses (%)	41	40	0.91
Sheep (%)	51	60	0.31
Other air pollution sources (%)			
*Temascal* wood-fired sauna	93	94	0.84
Kerosene lamp	21	14	0.34
Secondhand tobacco smoke	25	18	0.37

a Two-sided *p*-values from *t*-tests for continuous variables and chi-square tests for categorical variables.

b Values are mean ± SD for continuous variables.

c Asset index: sum of binary indicators for having a radio, television, and bicycle.

**Table 3:** Crude and adjusted between-group differences in SBP and DBP (mm Hg) associated with *plan-cha* compared with open fire use during the trial period.

	No. of subjects (measures)		Crude mean difference			Adjusted mean difference^a^		
	Control group	Intervention group	Estimate	95% CI	*p*-Value	Estimate	95% CI	*p*-Value
SBP	71 (111)	49 (115)	−2.3	−6.6 to 2.0	0.30	−3.7	−8.1 to 0.6	0.10
DBP	71 (111)	49 (115)	−2.2	−4.7 to 0.3	0.09	−3.0	−5.7 to −0.4	0.02

Measures refers to the total number of observations, which were repeated measures within subject on different days.

a Adjusted for age, BMI, daily average apparent temperature, rainy season, day of week, time of day, use of a *temascal* (wood-heated sauna), having household electricity, an asset index, ever smoking, and SHS exposure.

**Table 4:** Crude and adjusted within-subject differences in SBP and DBP (mm Hg) after the *plancha* echo-intervention compared with before.

	No. of subjects (measures)		Crude mean difference			Adjusted mean difference^a^		
	Trial period	Echo-intervention	Estimate	95% CI	*p*-Value	Estimate	95% CI	*p*-Value
SBP	55 (88)	55 (65)	−3.7	−6.0 to −1.4	0.002	−3.1	−5.3 to −0.8	0.01
DBP	55 (88)	55 (65)	−2.3	−3.8 to 0.9	0.003	−1.9	−3.5 to −0.4	0.01

Measures refers to the total number of observations, which were repeated measures within subject on different days.

a Adjusted for age, BMI, daily average apparent temperature, rainy season, day of week, time of day, use of a *temascal* (wood-heated sauna), having household electricity, an asset index, ever smoking, and SHS exposure.
